# Advances in Manufacturing and Characterization of Functional Polyesters

**DOI:** 10.3390/polym12122839

**Published:** 2020-11-29

**Authors:** Rafael Balart, Nestor Montanes, Octavio Fenollar, Teodomiro Boronat, Sergio Torres-Giner

**Affiliations:** 1 Technological Institute of Materials (ITM), Universitat Politècnica de València (UPV), Plaza Ferrándiz y Carbonell 1, 03801 Alcoy, Spain; nesmonmu@upvnet.upv.es (N.M.); ocfegi@epsa.upv.es (O.F.); tboronat@dimm.upv.es (T.B.); 2 Research Institute of Food Engineering for Development (IIAD), Universitat Politècnica de València (UPV), Camino de Vera s/n, 46022 Valencia, Spain

In the last few years, a remarkable growth in the use of functional polyesters has been observed. This trend comprises the development of aromatic polyesters derived either from renewable resources or recycling processes on account of the rapid depletion of fossil fuels and the cost of extracting polymers from petroleum. Furthermore, biodegradable aliphatic polyesters are also being rapidly developed due to pollution deriving from traditional plastics. The latter group includes both bio-based and petroleum derived polyesters that are biodegradable, that is, they can undergo biodisintegration under controlled compost soil or natural conditions. Moreover, blends and composites based on these novel polyesters represent a recurrent and cost-effective solution due to their good balance in terms of easy manufacturing, improved sustainability profile, and tailor-made performance. All these polyesters do not only contribute to sustainable development but they can also be functionalized to tailor their desired properties in terms of thermal and mechanical properties, barrier performance, biodegradation, and biocompatibility. For instance, copolymerization and the use of micro- and nanoparticles or reactive compatibilizers can widen the potential use of polyesters in both commodity and technical areas such as packaging, textiles, automotive, building and construction, and also in specialized fields such as tissue engineering and controlled release of drugs, electronics or shape-memory devices. The present Special Issue gathers a series of thirteen articles focused on the manufacturing and characterization of functional polyesters.

In terms of technical applications, the synthesis of functional materials is increasingly important for optical devices and electronics. In this regard, Jeong et al. [1] developed a novel copolyester for next-generation flexible devices in optics. To this end, authors synthesized by melt polymerization and subsequent solid-state polycondensation (SSP) a copolyester, named PCITN, based on 2,6-naphthalene dicarboxylic acid (NDA), terephthalic acid (TPA), 1,4-cyclohexanedimethanol (CHDM), and isosorbide (ISB). Polymerization carried out in two steps led to a PCITN copolyester having a high molecular weight (Mw = 68,900 g/mol), which was successfully shaped thereafter into films by extrusion at 290 °C. The resultant randomly-oriented PCITN films were preheated for 25 min and then tensile stretched and uniaxially cold drawn at 150 °C in machine direction (MD) at different draw ratios (λ = 1~4) to increase molecular chain orientation. The uniaxially oriented films showed high glass transition temperature (T_g_ of up to 140 °C) and Young’s modulus (E = 2.6 GPa), which was ascribed to the use of the low segmental mobility and high thermal stability of the building block ISB. The PCITN films also presented lower water absorption (0.54 wt.%) and birefringence (△n = 0.09) when compared with other conventional substrate polymers used for flexible electronics such as polyimide (PI), poly(ethylene 2,6-naphthalate) (PEN), and polyethylene terephthalate (PET). Copolymerization in two stages was also explored by Safari et al. [2] to develop random copolyesters with adjustable properties depending on their crystallinity. Authors synthesized poly(butylene succinate)-*ran*-poly(ε-caprolactone) (PBS-*ran*-PCL) copolyesters by transesterification/ring opening polymerization (ROP) reaction of 1,4-butanediol (BD), dimethyl succinate (DMS), and ε-caprolactone (CL) followed by polycondensation at reduced pressure. The PBS-*ran*-PCL copolyesters showed isodimorphic behavior with a controllable balance between comonomer inclusion and exclusion, where at some intermediate compositions the crystal lattice of each one of the components partially tolerate the presence of the other. In particular, the pseudo-eutectic point was found at 55 mol.% CL, where both PBS- and PCL-rich phases crystallized, whereas single PBS- and PCL-rich crystals were respectively formed at compositions based on lower and higher CL-unit molar contents. Furthermore, the phase crystallization of PBS-*ran*-PCL with pseudo-eutectic composition was successfully controlled by varying the isothermal crystallization temperature.

Only specifically developed functional polyesters can fulfill the specific challenges of the constantly developing biomedical field. At present, biodegradable aliphatic polyesters are being intensively studied as resorbable materials in bone tissue engineering, particularly for low-stress parts such as small orthopedic plates, rods or bone screws. Accordingly, these polyesters can develop scaffolds and biomaterial devices with desired geometries and special functionalities to attain osteoconductive properties. However, polyesters cannot satisfy most of the current mechanical requirements for bone fixation of load-bearing devices. In this regard, the use of micro- and nano-scale mineral particles with high hardness and different bioactives is very promising. For instance, nanoparticles of hydroxyapatite (nHAs) were used by Ivorra-Martinez et al. [3] to improve the mechanical performance of the poly(3-hydroxybutyrate-*co*-3-hydroxyhexanoate) [P(3HB-*co*-3HHx)] copolyester. It was observed that the incorporation of the osteoconductive nanofiller yielded balanced properties to the microbial copolyester in terms of strength and ductility, showing values of tensile modulus (E_t_) and elongation at break (ε_b_) ranging from approximately 1 up to 1.7 GPa and 6.5 to 19.4%, respectively. As a result, the P(3HB-*co*-3HHx)/nHA nanocomposites presented a closer mechanical performance to that found in the natural bone when compared to titanium (Ti) and alloys of metals such as stainless steel and cobalt-chrome (Co–Cr) alloys.

Another challenge that polyesters is facing in the biomedical area is related to their inadequate degradation rates, which would increase the risk of requiring additional chirurgical interventions, eventually causing adverse tissue reactions or even infections. In this context, Torres et al. [4] also employed nHAs and halloysite nanotubes (HNTs) to increase the surface wettability of hydrophobic and hydrophilic sets of poly(ε-caprolactone) (PCL), polylactide (PLA), and their blends as well as poly(2-hydroxyethyl methacrylate) (PHEMA) and its copolymer with ethyl methacrylate (EMA), that is, poly(2-hydroxyethyl methacrylate-*co*-ethyl methacrylate) P(HEMA-*co*-EMA). Authors demonstrated that both the blending of PCL with PLA and the incorporation of nHAs and HNTs provided hydrophilic units and decreased the crystallinity of PCL, favoring the accessibility of water molecules to the ester linkages. Therefore, in comparison with the unfilled PCL/PLA blend, mass loss increased up to 48% after incubation for 12 weeks in phosphate buffered saline (PBS) during the evaluation of their degradation rate. Consequently, the mechanical properties of the polyesters decreased above 60% after the incubation time due to the higher hydrolytic cleavage achieved. Similarly, Zhao et al. [5] developed nanocomposites of PLA with 1 wt.% of nanoparticles and whiskers of magnesium oxide (MgO) that were chemically modified with stearic acid. The in vitro degradation of the nanocomposites was analyzed by PBS soaking for up to 12 months, showing that the addition of the stearic acid-modified MgO accelerated the PLA’s water uptake rate, especially for the whiskers. Furthermore, the dissolution of MgO through the neutralization of the acidic product of the PLA degradation contributed to regulate the pH value of PBS. The in vivo results of the histological morphologies attained with the nanocomposites further suggested that the presence of MgO can improve bone repair since, when dissolving, it releases magnesium ions (Mg^2+^) that can activate a variety of enzymes that promote the synthesis of proteins. In addition, PLA-based materials can also serve as a drug carrier for controlled release applications. Micro- and nanoencapsulation using HNTs is promising to deliver different active and bioactive compounds such as antioxidants, antimicrobials, antibiotics, or growth factors (GFs). In this regard, Montava-Jorda et al. [6] developed PLA composites with HNTs loadings of 3, 6, and 9 wt.%. Authors found that water uptake of PLA increased due to the hydrophilic nature of the nanotubes, which offer a high surface area with hydroxyl (–OH) groups, and thus can favor the degradation rate of the resultant wound dressings.

One of the most exciting areas of functional polyesters is the development of sound absorbing materials. It has been demonstrated that, for instance, nonwoven structures of polyester fibers are potential candidates in noise reducing panels. The research article of Yang et al. [7] estimated the non-acoustic parameters of high-loft nonwoven panels made of a commercial polyester blend composed of 45 wt.% staple polyester, 30 wt.% hollow polyester, and 25 wt.% bicomponent polyester provided by the Technical University of Liberec (Liberec, Czech Republic). The panels, with densities ranging from 16.93 to 45.56 kg/m^3^, were manufactured by perpendicular laying technology and were tested by means of the Bayesian reconstruction procedure, implementing the Johnson-Champoux-Allard-Lafarge model and Markov chain Monte Carlo optimization technique. The inversed method showed that the polyester-based panels were homogeneous along with the panel thickness, presenting the same inferred tortuosity. Mean relative differences of airflow resistivity and porosity of 0.019 and 0.004 were respectively attained, which mostly affected the thermal characteristic length. In the textile area, linen (*Linum usitatissimum*) is among the most usable and profitable plants. One of its processing by-products is flax fiber (FF), which can also be applied to replace glass fiber (GF) as a suitable reinforcement in advanced composites of polyesters for several engineering applications such as panel boards and insulation panels. However, lignocellulosic fibers habitually show a poor interfacial adhesion with hydrophobic polyester matrices such as PLA. In this context, Agüero et al. [8] evaluated the influence of different compatibilization strategies on the performance of PLA/FF composites. In particular, the compatibilization routes consisted of silanization with (3-glycidyloxypropyl) trimethoxysilane (GPTMS) and reactive extrusion (REX) with a commercial random copolymer of poly(styrene-*co*-glycidyl methacrylate) (PS-*co*-GMA, Xibond™ 920, Polyscope, Geleen, The Netherlands), a multi-functional epoxy-based styrene-acrylic oligomer (ESAO, Joncryl ADR 4368^®^, BASF S.A., Barcelona, Spain), and maleinized linseed oil (MLO). Among the tested routes, the petroleum derived ESAO yielded the highest mechanical resistance and toughness improvement and also the highest thermal stability due to the chain-extension or cross-linking effect on PLA, whereas the most ductile green composites were attained with MLO by plasticization.

Focusing on the packaging field, the PLA biopolyester is widely used for food containers (e.g., food films and trays) or disposable articles (e.g., straws and cutlery). However, it habitually results in extremely brittle materials with low ductility and toughness. To improve the impact properties of PLA, Lascano et al. [9] incorporated an oligomer of lactic acid (OLA) in the contents of 5–20 wt.%. At a loading of 15 wt.% OLA, it was observed a percentage increase of nearly 171% in the impact strength of PLA. Furthermore, for low deformation angles, the OLA-containing PLA samples successfully recovered over 95% of their original shape, while for the highest angles they still reached a recovery of approximately 70%. In packaging applications, the development of eco-friendly or green composites by the valorization of agrofood waste also shows several benefits including the enhancement of the environmental profile, improved biodegradability, lightweight, or cost reduction. Among the wide variety of agricultural wastes and food processing by-products, almond (*Prunus amygdalus* L.) shell powder (ASP) was used by Ramos et al. [10] as the filler for a commercial biopolyester blend (INZEA F2^®^, Nurel, Zaragoza, Spain) at 10 and 25 wt.%. In this study, the lignocellulosic filler was first subjected to two grinding levels, yielding sizes of 125–250 and 500–1000 μm, and MLO and low-functionality ESAO (Joncryl ADR 4400^®^, BASF S.A.) were added as compatibilizers. Authors reported that ASP successfully improved the biodisintegration rate of PLA under composting conditions, while full disintegration was obtained after 90 and 28 days for the green composites containing 10 and 25 wt.% ASP, respectively. Authors concluded that the presence of ASP at high contents produced a significant discontinuity in the polyester matrix, facilitating water penetration and favoring the growth of microorganisms responsible for biodisintegration.

Finally, disposal of waste polyester-based materials has become an urgent environmental problem in the last decades. For instance, PET, which is widely used worldwide in bottles for water and beverages, food trays or fibers for textiles, produces a plastic waste that is neither biodegradable nor compostable, and recycling currently represents the only solution. Although the post-consumer uses of recycled polyethylene terephthalate (r-PET) streams has increased, it is currently limited to low contents in mixed formulations with virgin PET due to the weakening of its physical-mechanical properties as a result of the M_W_ reduction during processing derived from the cleavage of the polyester chains. Thus, the use of nanostructured additives and/or reactive additives has emerged as a novel route to deal with this technical issue. In the study of Dominici et al. [11], anhydrous calcium terephthalate anhydrous salts (CATAS), a nanometric metal organic framework consisting of calcium ions (Ca^2+^) as metal clusters coordinated to TPA as organic ligand, were developed and incorporated by melt processing into rPET at different two levels of loadings, that is, 0.1–1 wt.% and 2–30 wt.%. Authors found that 0.4 wt.% of CATAS led to the formation of a rigid amorphous fraction due to the aromatic interactions (π−π conjugation) between the rPET matrix and Ca-based salts, which was located at the rPET/CATAS interface. In contrast, tangible changes were observed below this threshold, whereas a restriction of rPET/CATAS molecular chains mobility was detected above 0.4 wt.% CATAS due to the formation of percolation networks with hybrid mechanical characteristics. In another study, Montava-Jorda et al. [12] melt-mixed at 15–45 wt.% partially bio-based polyethylene terephthalate (bio-PET) with r-PET flakes that were obtained from pre-consumer waste streams of the food-use bottle industry and PS-*co*-GMA at 1–5 parts per hundred resin (phr) of polyester blend. For the polyester blend containing 45 wt.% of r-PET, the addition of 5 phr of the reactive compatibilizer led to an enhancement in the elongation-at-break value from 10.8 to 378.8%, whereas impact strength also increased from 1.84 to 2.52 kJ/m^2^. It was concluded that, due to a chain-extension mechanism based on the reaction of the–OH and carboxyl (–COOH) terminal groups of both bio-PET and r-PET chains with the multiple groups of glycidyl methacrylate (GMA) present in PS-*co*-GMA, branched and larger interconnected macromolecules were formed and contributed to improve the ductile performance of the polyester blends. Additionally, in terms of improving the mechanical or secondary recycling of polyesters with other polymers, Jorda et al. [13] reported binary blends of bio-PET with polyamide 1010 (PA1010), a fully bio-based polyamide (bio-PA), compatibilized with PS-*co*-GMA. Authors reported that the addition of 30 wt.% of PA1010, which provides a final renewable content of nearly 50 wt.% in the polymer blend, yielded an immiscible droplet-like structure in which PA1010 droplets are embedded in the bio-PET matrix. However, the intrinsic stiffness of the biopolyester was improved by the bio-PA also the addition of PS-*co*-GMA at 3 phr, which induced a remarkable reduction of the droplet size from approximately 4 to 1 mm. According to these studies, secondary recycling of bio-based but non-biodegradable polyesters can be feasible from environmental and economic points of view.

From the above, the Special Issue *Advances in Manufacturing and Characterization of Functional Polyesters* published in *Polymers,* brings together a broad range of research works dealing with functional polyesters to update the “state-of-the-art” knowledge in this field. Functionalization of polyesters was successfully achieved by means of copolymerization, the use of additives at micro- and nano-scale or reactive compatibilizers. The resultant materials can find potential uses in technical applications, such as optical devices and electronics, in the biomedical field for tissue engineering and controlled release of drugs, or as sound absorbing panels, textiles and apparel, advanced composites, and sustainable food packaging.
